# Supplementary material for: Severe hypoxia drives loss of ST6GAL1-mediated α2,6-sialylation in the epicardial secretome impairing angiogenic activity
Source: Biol Open. 2026 Apr 29;15(4):bio062479. doi: 10.1242/bio.062479 (PMC13225208; doi:10.1242/bio.062479)
Supplement: Supplementary information [file biolopen-15-062479-s1.pdf]

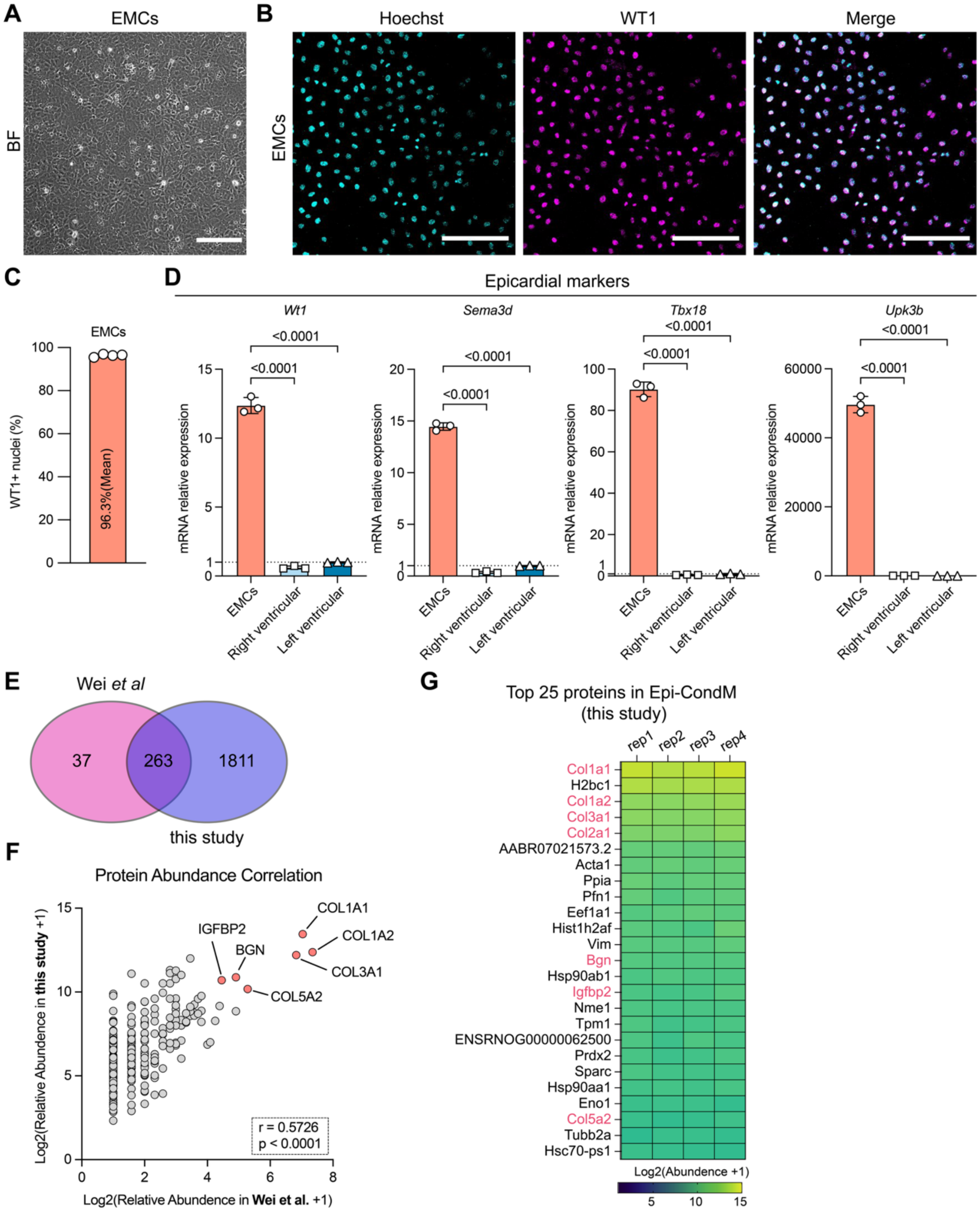

Fig. S1. Characterization of rat epicardial mesothelial cells (EMCs) and proteomic profiling of epicardial conditioned media (Epi-CondM).

(A) Brightfield (BF) microscopy image of EMCs prior to Epi-CondM preparation (~98% confluence). Scale bar, 200 µm.

(B) Immunofluorescence staining of WT1 (Wilms' tumor 1), a specific epicardial marker, in EMCs. Nuclei were counterstained with Hoechst. Images were acquired using a confocal microscope. Scale bar, 200 µm.

Biology Open • Supplementary information

(C) Quantitative analysis of the ratio of WT1-positive EMCs.

(D) Expression levels of epicardial markers (*Wt1*, *Sema3d*, *Tbx18*, and *Upk3b*) in EMCs compared to rat right ventricular and rat left ventricular tissues, as determined by qPCR. Gene expression was normalized to *Gapdh* and presented as fold change relative to left ventricular tissues. Primer sequences used for qPCR are provided in **Table S1**.

(E) Venn diagram showing the overlap of proteins identified in Epi-CondM between this study and the dataset reported by Wei *et al.* 2015.

(F) Correlation analysis of protein abundance for the commonly identified proteins between this study and Wei et al. The x-axis indicates relative protein abundance reported by Wei et al., and the y-axis indicates relative abundance measured in this study (log_2-transformed values). Selected proteins are labeled. The Pearson correlation coefficient (r) and p value are shown.

(G) Heatmap showing the top 25 most abundant proteins detected in Epi-CondM across biological replicates (rep1–rep4). Color intensity represents log_2-transformed protein abundance.

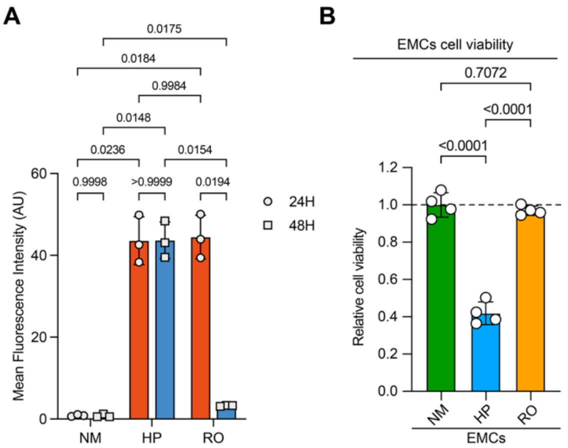

Fig. S2. Intercellular oxygen level and cell viability in EMCs under normoxia (NM), hypoxia (HP), and reoxygenation (RO) conditions.

(A) Quantification analysis of hypoxia (low oxygen level) in EMCs. Bar charts display the mean fluorescence intensity (AU) obtained from the Image-iT™ hypoxia reagent within EMCs at 24 hours and 48 hours under normoxia (NM), hypoxia (HP), and reoxygenation (RO) conditions. The increased fluorescence intensity reflects low intracellular oxygen levels (hypoxia).

(B) Cell viability of EMCs under NM, HP, and RO conditions, assessed by CCK-8 assay at 48 hours.

Biology Open • Supplementary information

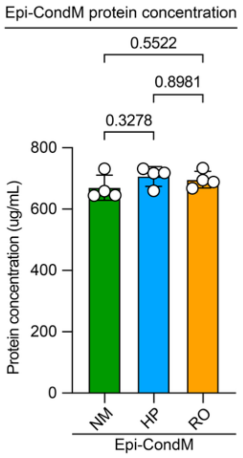

Fig. S3. Total protein concentration in epicardial conditioned media (Epi-CondM). Protein concentration in stressed Epi-CondM (NM, HP, and RO) measured by bicinchoninic acid (BCA) assay.

Biology Open • Supplementary information

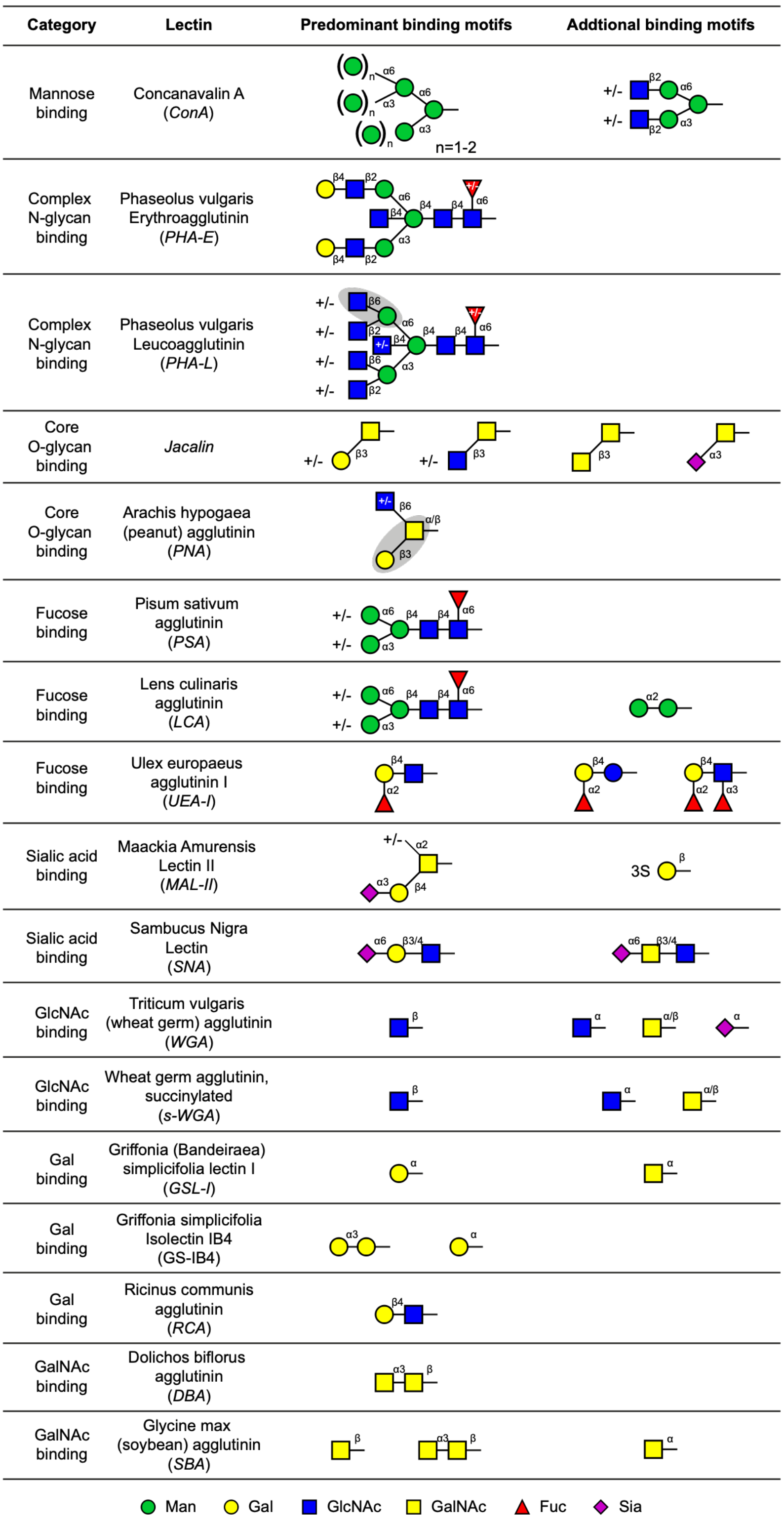

Fig. S4. Glycan-binding specificity of the lectin panel used in this study.

The binding specificities of 17 lectins were annotated according to their predominant glycan recognition motifs. Glycan structures are represented using the Symbolic Nomenclature for Glycans (SNFG): mannose (Man, green circles), galactose (Gal, yellow circles), N-acetylglucosamine (GlcNAc, blue squares), N-acetylgalactosamine (GalNAc, yellow squares), fucose (Fuc, red triangles), and sialic acid (Sia, purple diamonds)

Biology Open • Supplementary information

Table S1. Primer sequences for qPCR analysis of epicardial marker gene expression in rat EMCs.

Primer	Sequence (5'-3')
Wt1-F	CTCGCTCAGACCAGCTCAAA
Wt1-R	GCTGAAGGGCTTTTCACTTGTT
Sema3d-F	CTCTCCTTGCACAGATGCGA
Sema3d-R	GGCGTCTAGCTCGCCTTTT
Tbx18-F	TCTTCACAACTGTCACTGCCT
Tbx18-R	GCTTCCAAACCCATTCTGTTCC
Upk3b-F	AACTTCACAGAGACGCCTGC
Upk3b-R	TGGGGTTCTTCCCTTGGTGG
Gapdh-F	TATTGCAACGCTCCCCTCC
Gapdh-R	CTGGAAGATGGTGATGGGTT

Table S2. Primer sequences for qPCR validation of *St6gal1* knockdown in EMCs.

Primer	Sequence (5'-3')
St6gal1-F	CTCCGGCATGCTGGGTATCA
St6gal1-R	CGTCTTGCGCTTGGATGGGA
Gapdh-F	TATTGCAACGCTCCCCTCC
Gapdh-R	CTGGAAGATGGTGATGGGTT

Biology Open • Supplementary information
